# The PBMC transcriptome profile after intake of oxidized versus high-quality fish oil: an explorative study in healthy subjects

**DOI:** 10.1186/s12263-016-0530-6

**Published:** 2016-05-31

**Authors:** Mari C. W. Myhrstad, Inger Ottestad, Clara-Cecilie Günther, Einar Ryeng, Marit Holden, Astrid Nilsson, Kirsti W. Brønner, Achim Kohler, Grethe I. A. Borge, Kirsten B. Holven, Stine M. Ulven

**Affiliations:** 1 Department of Health, Nutrition and Management, Faculty of Health Sciences, Oslo and Akershus University College of Applied Sciences, P.O. Box 4, St. Olavs plass, 0130 Oslo, Norway; 2 Department of Nutrition, Institute for Basic Medical Sciences, University of Oslo, P.O. Box 1046, Blindern, 0317 Oslo, Norway; 3 Norwegian Computing Center, 0314 Oslo, Norway; 4 Department of Cancer Research and Molecular Medicine, Norwegian University of Science and Technology, 7489 Trondheim, Norway; 5 Nofima AS, Norwegian Institute of Food, Fisheries and Aquaculture Research, PB 210, Aas, N-1431 Norway; 6 TINE SA, Centre for Research and Development, P.O. Box 7, Kalbakken, 0902 Oslo, Norway; 7 Department of Mathematical Sciences and Technology (IMT), Norwegian University of Life Sciences, 1432 Ås, Norway; 8 Norwegian National Advisory Unit on Familial Hypercholesterolemia, Department of Endocrinology, Morbid Obesity and Preventive Medicine, Oslo University Hospital Rikshospitalet, P.O Box 4950, Nydalen, Oslo, Norway

**Keywords:** Oxidized fish oil, n-3 fatty acids, PBMCs, Transcriptome, Human intervention

## Abstract

**Background:**

Marine long-chain polyunsaturated fatty acids are susceptible to oxidation, generating a range of different oxidation products with suggested negative health effects. The aim of the present study was to utilize sensitive high-throughput transcriptome analyses to investigate potential unfavorable effects of oxidized fish oil (PV: 18 meq/kg; AV: 9) compared to high-quality fish oil (PV: 4 meq/kg; AV: 3).

**Methods:**

In a double-blinded randomized controlled study for seven weeks, 35 healthy subjects were assigned to 8 g of either oxidized fish oil or high quality fish oil. The daily dose of EPA+DHA was 1.6 g. Peripheral blood mononuclear cells were isolated at baseline and after 7 weeks and transcriptome analyses were performed with the illuminaHT-12 v4 Expression BeadChip.

**Results:**

No gene transcripts, biological processes, pathway or network were significantly changed in the oxidized fish oil group compared to the fish oil group. Furthermore, gene sets related to oxidative stress and cardiovascular disease were not differently regulated between the groups. Within group analyses revealed a more prominent effect after intake of high quality fish oil as 11 gene transcripts were significantly (FDR < 0.1) changed from baseline versus three within the oxidized fish oil group.

**Conclusion:**

The suggested concern linking lipid oxidation products to short-term unfavorable health effects may therefore not be evident at a molecular level in this explorative study.

**Trial registration:**

ClinicalTrials.gov, NCT01034423

**Electronic supplementary material:**

The online version of this article (doi:10.1186/s12263-016-0530-6) contains supplementary material, which is available to authorized users.

## Background

Fish oil (FO) is associated with reduced risk of coronary heart disease (CHD) and CHD deaths [1, 2]; thus, the use of supplements containing n-3 fatty acids are recommended for CHD prevention among those who do not eat fish in accordance with the food recommendations [3–5]. Long-chain polyunsaturated fatty acids (LCPUFAs), including n-3 fatty acids in marine oils, are susceptible to oxidation, and consequently, lipid oxidation products are generated [6–8]. For several years, the voluntary monographs by the Global Organization for eicosapentaenoic acid (EPA) and docosahexaenoic acid (DHA) Omega-3s (GOED) have served as the quality standard of the industry, giving recommendations for the content of lipid oxidation products, such as a maximum peroxide value (PV) of 5 meq/kg (milliequivalents per kilogram) and a maximum anisidine value (AV) of 20 [9]. Other monographs exists, such as the European pharmacopeia recommending a PV at 5 or 10 meq/kg and an AV at 30 or 15, depending on the content of marine n-3s. The quality of the FO, in terms of the oxidative status, can be camouflaged by encapsulation, additives, and flavorings [10], and a variation in the oxidative status of n-3 supplements and vegetable oils has been reported [11–13].

Concerns related to a regular consumption of oxidized marine oils and negative health effects have been raised [10, 14, 15]. If lipid oxidation products from the diet are absorbed in humans, these highly reactive compounds may cause oxidative damage to macromolecules such as proteins, carbohydrate, DNA, and lipids thereby altering their function [16]. Highly reactive lipid compounds may also act as signaling molecules that alter gene functions and thereby influence health via cellular sensing mechanisms [16].

Cell studies have indicated that lipid oxidation products are involved in pathophysiological processes due to their reactivity [7, 17–19], and oxidized n-3 fatty acids have been shown to increase the antioxidant defense system and to enhance endoplasmic reticulum (ER) stress in human intestinal Caco-2/TC7 cells [20]. Some animal studies show serious biological effects from the intake of oxidized lipids [21], but there are few studies investigating the intake of oxidized lipids in concentrations more similar to daily life. However, mice fed on a high-fat diet containing weakly oxidized n-3 fatty acids for 8 weeks increased the plasma level of n-3 oxidation products and inflammatory markers [20]. The intake of oxidized vegetable oils has been shown to increase the postprandial level of plasma lipid oxidation products and markers related to coronary vascular disease in humans [22–26]. Discrepancies exist, and we have previously reported that the intake of oxidized fish oil for 7 weeks did not change the plasma levels of peroxidation products of LCPUFAs, serum level of oxidized LDL-cholesterol, or selected markers of oxidative stress and inflammation [27, 28]. However, lipid oxidation generates a variety of reactive compounds [6–8], and no single biomarker reflects the total in vivo lipid oxidation and oxidative stress status. In addition, the circulating oxidative stress markers may not be sensitive enough to reflect a local change in the cellular oxidative stress status. Other approaches including more sensitive techniques to accurately measure the influence of lipid oxidation products at a molecular level are therefore required.

Transcriptome analyses examine the mRNA transcript level in a given cell population and reflect the genes that are actively expressed at a given time. Changes in the transcriptome profile occur prior to changes in protein levels. Transcriptomics has therefore shown to be a valuable and sensitive technique measuring early changes related to a dietary challenge [29, 30]. The peripheral blood mononuclear cells (PBMCs) include monocytes and lymphocytes. In the circulation, PBMCs are exposed to environmental factors and metabolic tissues and may therefore reflect systemic health. It is well known that dietary factors modulate the gene expression in PBMCs [31–35]. In the same intervention trials as presented here, we have previously shown that the intake of high-quality FO modulated the transcriptional profile in human PBMCs and changes in the gene transcripts related to cell cycle, apoptosis, and ER stress were observed [36]. The potential of transcriptome analyses to detect early changes caused by a nutritional challenge encourages us to further investigate whether the intake of oxidized fish oil (oxFO) could alter the gene expression profile in PBMCs compared to high-quality FO in healthy subjects.

## Methods

### Subjects

**Fig. 1:**
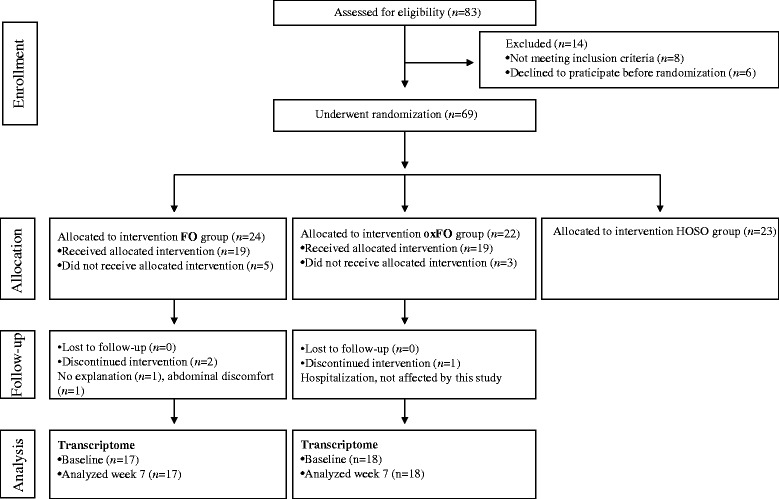
Flow chart of the study showing subjects enrolled, lost during follow-up, and number of subjects included in the statistical analysis at the baseline and after 3 weeks of fish oil supplementation. *FO group* fish oil group, *oxFO* oxidized fish oil group, *HOSO* high-oleic sunflower oil group

The intervention study took place at Akershus University College, Norway, between September and December 2009. Thirty-five young healthy subjects (10 men and 25 women, aged 28.0 ± 8.1 years) with body mass index (BMI) and serum lipids within the normal range were included. Subjects enrolled in the present study are shown in Fig. 1. The detailed description of the protocol, participant recruitment and enrolment, inclusion and exclusion criteria, and compliance are published in detail elsewhere [28].

### Study design

This study was a part of a randomized controlled double-blinded three-arm parallel group study, designed to investigate the health effects from the intake of fish oil with different oxidative quality [28]. In the present study, data from two of the intervention arms are included, as shown in Fig. 1. All subjects received 16 capsules each day, containing 8 g FO per day, of which 1.6 g was n-3 fatty acids (0.7 g/d EPA + 0.9 g/d DHA). Both FOs were originating from the same batch of cod liver oil (Gadidae sp., TINE EPA/DHA Oil 1200) provided by TINE SA (Oslo, Norway), and they were identical except for the oxidative status level. The intervention group received either oxidized oxFO (PV, 18 meq/kg; AV, 9; *n*, 18) or FO (PV, 4 meq/kg; AV, 3; *n*, 17) (Table 1). Further characterization of the encapsulated oils have previously been published [28]. The subjects were instructed to take the capsules with food (minimum two meals). Blood samples were collected at baseline and after 3 and 7 weeks of intervention. The blood samples after 7 weeks are utilized in the current study. Prior to the baseline visit, the subjects underwent a 4-week marine n-3 fatty acid washout period. During the first 3 weeks of the intervention period, the subjects conducted a fully controlled isoenergetic diet. The food items given did not contain any marine n-3 fatty acids, and the food provided has previously been described in detail elsewhere [28]. The intake of dietary supplements and marine n-3 fatty acids was not allowed during the intervention period.

**Table 1:** Characterization of the encapsulated oil

	oxFO	FO
Omega-3 fatty acids		
EPA (20: 5n-3) (g/100 g)	9.1	9.0
DHA (22: 6n-3) (g/100 g)	11.2	11.1
DPA (22 : 5n-3) (g/100 g)	1.1	1.1
Oxidation level		
PV (meq/kg)	18	4
AV	9	3
TOTOX (2 × PV + AV)	45	11
Volatile oxidation products of omega-3 FA		
Pentanal (μg/100 g)	137.8	6.2
1-penten-3-ol (μg/100 g)	132.4	12.8

*oxFO* oxidized fish oil, *FO* fish oil, *EPA* eicosapentaenoic acid, *DHA* docosahexaenoic acid, *DPA* docosapentaenoic acid, *PV* peroxide value, *AV* anisidine value, *TOTOX* total oxidation

### PBMCs and RNA isolation

After blood collection, the PBMCs were isolated by using the BD Vacutainer Cell Preparation tubes according to the manufacturer’s instructions (Becton, Dickinson and Company, NJ 07417, USA). Pellets were frozen and stored at −80 °C for further RNA isolation.

Total RNA was isolated from all PBMC samples using the RNeasy Mini Kit (Qiagen) with lysis buffer-added β-mercaptoethanol according to the manufacturer’s instructions and stored at −80 °C. RNA quantity and quality measurements were performed using the ND 1000 Spectrophotometer (Saveen & Werner AB, Limhamn, Sweden) and Agilent Bioanalyser (Agilent Technologies Inc., CA 95051, USA), respectively. One sample had a RNA integrity number (RIN) score of 3.3, and all other samples were above 8 (mean = 9.6). Nanodrop analysis did not indicate any contamination in the RNA samples. The total numbers of monocytes and lymphocytes were measured in EDTA plasma at a routine laboratory (Fürst Medical Laboratory, Oslo, Norway).

### Microarray hybridization

Labeled extracts were prepared using the Illumina TotalPrep RNA Amplification Kit (Illumina Inc., CA 92122, USA) according to manufacturer’s instructions. The labeled extracts were hybridized on an Illumina HumanHT-12 v4 Expression BeadChip and scanned on an Illumina HiScan microarray scanner (Illumina Inc., CA 92122, USA). Illumina GenomeStudio was used to transform bead-level data to probe-level intensity values and statistics, which were exported raw (unfiltered, non-normalized) for bioinformatic analysis. After hybridization and scanning, a manual quality control step was performed, looking at density plots and hierarchical clustering of raw probe densities. All samples, including the one with RIN < 8 were displaying good characteristics and included in further analysis.

### Microarray data analyses

The Illumina intensities were quantile-normalized. To improve statistical power, probes without a detectable expression (detection *p* value > 0.01) in at least 10 % of the samples were discarded from further analysis. From the 48,000 probes presented on the HumanHT-12 v4 microarray, 21,236 were defined as expressed in the PBMCs and included in the analyses. Changes in gene expression were obtained by calculating log_2_ ratios between the baseline and after 7-week intensities, and the two intervention groups were compared with regard to this ratio. Differentially expressed genes between groups were identified using the Linear Models for Microarray (Limma) data [37] package from Bioconductor (http://www.bioconductor.org) performed with the R software. For the within-group analyses, the log_2_ intensities at 7 weeks and baseline were compared and differentially expressed genes within each group were identified using Limma.

Genes with a nominal *p* value < 0.05 were defined as differentially regulated and subjected to further gene ontology, pathway, and network analyses using the Database for Annotation, Visualization and Integrated Discovery (DAVID) software tool version 6.7 (http://david.abcc.ncifcrf.gov) and MetaCore (GeneGo, division of Thomson Reuters, St. Joseph, MI, USA). For the DAVID analyses, the list of differential regulated genes was compared to a reference list of Homo sapiens and biological processes containing more than 10 genes and a false discovery rate (FDR) *q* value < 0.05 were considered significantly modulated. Pathways and networks identified in MetaCore with a FDR *q* value < 0.05 were considered significantly modulated.

Gene Set Enrichment Analyses (GSEA) (http://www.broad.mit.edu/gsea/) were applied on the genes defined as expressed in PBMCs (21,236) for comparison of the transcriptome profile between the two groups. The gene sets in the collection of C2 canonical pathways (cps) and C5 biological process (bp) from Molecular Signatures Database (MSigDB v5.0) were used separately. In addition, six gene set collections associated with oxidative stress and cardiovascular disease were created using a gene set browser (MSigDB v5.0) on the Broad Institute website (http://software.broadinstitute.org/gsea/msigdb/search.jsp) using the keywords stepwise: “oxidative AND stress,” “oxidative AND damage,” “hypoxi*,” “cardiovascular*,” “immune AND response,” and “inflammation”. Permutation (1000) was performed on phenotypes, and gene sets were defined as significantly changed when FDR *q* value < 0.25 as recommended for explorative analyses by the Broad Institute (http://www.broad.mit.edu/gsea/). The minimum information about a microarray experiment (MIAME) standards [38] were followed in the analysis and storage of data.

## Results

The subjects included in the study were young and middle-aged adults (28.0 ± 8.1 years), and the characteristics of the subjects have previously been described [28]. In short, no differences in age, BMI, or serum lipids were observed between the oxFO (*n* = 18) and the FO groups (*n* = 17) at baseline or after 7 weeks of intervention (Table 2). Additionally, no differences in plasma fatty acids, including the level of long-chained n-3 fatty acids, were observed between the two groups after the intervention period as previously described [28]. The number of monocytes and lymphocytes were the same between the two groups at baseline and after 7 weeks of intervention (data not shown).

**Table 2:** Baseline characteristic and serum blood values at baseline

	FO (*n* 17)		oxFO (*n* 18)		
	Baseline		Baseline		
	Mean/median	SD/25–75 pers	Mean/median	SD/25–75 pers	*p*
Male/female (*n*)	5/12		5/13		
Age (year)	25	23–32	22	21–28	0.202
BMI (kg/m^2^)	22.1	2.5	22.2	1.7	0.849
Total-C (mmo/l)	4.6	0.8	4.7	0.9	0.646
LDL-C (mmol/l)	2.5	0.8	2.7	0.8	0.431
HDL-C (mmol/l)	1.5	0.3	1.4	0.4	0.598
TG (mmol/l)	0.8	0.7–0.9	0.9	0.5–1.5	0.921

Data are presented as mean and standard deviation (SD) or median (25–75 percentile), and differences between the groups were calculated using independent-samples *t* test or Mann-Whitney *U* test. *p* values <0.05 were considered significant

*oxFO* oxidized fish oil, *FO* fish oil, *Total-C* total cholesterol, *LDL-C* low-density lipoprotein cholesterol, *HDL-C* high-density lipoprotein cholesterol, *TG* triglycerides

### Gene expression profiling in PBMCs

Microarray hybridization was performed on RNA from PBMCs collected at baseline and after 7 weeks of supplementation with oxFO (*n* = 18) or FO (*n* = 17).

The differences in gene expression between the two groups were determined by a moderated *t* test (Limma), by comparing the relative change from baseline to 7 weeks of intervention among the 21,236 expressed genes. In total, 402 gene transcripts were found to be differentially expressed between the two intervention groups (*p* < 0.05) (Additional file 1: Table S1). However, no gene transcripts were significantly differentially expressed (FDR *q* value <0.1) between the two groups when adjusting for multiple testing (Additional file 1: Table S1). To identify differences across the two intervention groups related to biological processes, pathways, and networks, we analyzed functional relationships among the 402 differentially expressed gene transcripts obtained with Limma (*p* < 0.05). There was no significantly regulated biological processes, pathways, or networks between the two groups after 7 weeks using the software tools DAVID or MetaCore (FDR *q* value < 0.05, data not shown).

GSEA was used to test whether groups of genes involved in the same biological process or pathway were changed after intake of oxFO compared to FO among all the 21,236 defined expressed genes. We could not detect any differences between the two groups when using the C2 cp or the C5 bp gene set collection (MSigDB v5.0) (FDR *q* value < 0.25) (Additional files 2 and 3: Table S2 and S3). In addition, GSEA was used to test whether gene sets associated with oxidative stress, inflammation, and cardiovascular disease were differently changed during the intervention in the oxFO group compared to the FO group. However, these gene sets were not significantly changed in the two FO groups (Table 3).

**Table 3:** GSEA analyses. Gene sets associated with oxidative stress and cardiovascular diseases created using a gene set browser (Molecular Signatures Database v5.0)

GSEA gene set collection^a^	# gene sets in collection^b^	Regulated gene sets oxFO vs FO (FDR *q* value < 0.25)
oxidative AND stress	46	0
oxidative AND damage	5	0
hypoxi*	65	0
cardiovascular*	13	0
immune AND response	147	0
inflammation	29	0

*#* number of gene sets, *FDR* false discovery rate

* indicates truncated search words

^a^Collections of gene sets were obtained using a gene set browser from the Broad Institute website http://software.broadinstitute.org/gsea/msigdb/search.jsp by the listed keywords

^b^Number of gene sets passing the gene set size filter (min 10 genes, max 500 genes)

A moderated *t* test (Limma) was used to identify regulated gene transcripts within the two intervention groups separately, by comparing the transcript level at 7 weeks to the level at baseline. In total, 345 and 667 gene transcripts (*p* < 0.01) among the defined expressed genes were regulated within the oxFO or FO group after 7 weeks, respectively. Of these, 3 (oxFO) and 11 (FO) gene transcripts were significantly regulated when adjusting for multiple testing (FDR *q* value <0.1) (Table 4). Two of the gene transcripts (CD55 and SNORD13) were overlapping and significantly downregulated in both groups. BAK1 mRNA was significantly increased from the baseline within the oxFO group only. In addition, the gene transcripts shown in Table 2 were regulated in the same direction within the two groups after 7 weeks. To explore the biological processes modulated within each intervention group, the lists of 345 and 667 changed gene transcripts were subjected to functional analyses separately. Several biological processes were significantly modulated within each intervention group (Table 5). Modulated biological processes after 7 weeks within the oxFO group and FO group were related to translation elongation, translation, and apoptosis, whereas only cell cycle was modulated after intake of oxFO (Table 5).

**Table 4:** Significantly changed gene transcripts identified with Limma (FDR *q* value < 0.1) within the oxFO or FO group after 7 weeks of intervention

	FC within groups from baseline		FDR *q* value	
Gene	oxFO	FO	oxFO	FO
CD55	0.83	0.85	0.00	0.01
SNORD13	0.63	0.60	0.04	0.01
VNN2	0.90	0.80	0.59	0.01
SNORA12	0.82	0.70	0.59	0.03
RSBN1L	0.92	0.88	0.59	0.03
VNN2	0.87	0.77	0.62	0.04
LOC80054	1.05	1.09	0.62	0.09
POLR1D	0.96	0.90	0.66	0.09
SERPINB9	0.98	0.85	0.92	0.09
RAB11FIP2	0.99	0.90	0.92	0.09
SNORD12C	0.91	0.88	0.59	0.10
BAK1	1.13	1.08	0.04	0.24

*oxFO* oxidized fish oil, *FO* fish oil, *FC* fold change from baseline, *FDR* false discovery rate

**Table 5:** Enriched biological processes within the oxFO and FO groups after 7 weeks of intervention among the regulated genes obtained with Limma analysis (*p* < 0.05)

Enriched BP oxFO	Fold enrichment	FDR %
GO:0006414~translational elongation	9.52	0.00
GO:0007049~cell cycle	2.48	0.02
GO:0006412~translation	3.49	0.03
GO:0000280~nuclear division	4.08	0.07
GO:0007067~mitosis	4.08	0.07
GO:0000087~M phase of mitotic cell cycle	4.01	0.08
GO:0048285~organelle fission	3.92	0.10
GO:0000278~mitotic cell cycle	3.12	0.11
GO:0022402~cell cycle process	2.38	0.85
GO:0051301~cell division	3.04	1.19
GO:0022403~cell cycle phase	2.63	1.23
GO:0042981~regulation of apoptosis	1.99	2.62
GO:0043067~regulation of programmed cell death	1.97	2.98
GO:0010941~regulation of cell death	1.97	3.13
GO:0000279~M phase	2.73	3.15
GO:0051726~regulation of cell cycle	2.71	3.34
GO:0051338~regulation of transferase activity	2.59	3.37
Enriched BP FO		
GO:0006414~translational elongation	9.22	0.00
GO:0006412~translation	3.83	0.00
GO:0006916~anti-apoptosis	3.80	0.00
GO:0043066~negative regulation of apoptosis	2.53	0.14
GO:0043069~negative regulation of programmed cell death	2.49	0.18
GO:0060548~negative regulation of cell death	2.48	0.18
GO:0043067~regulation of programmed cell death	1.88	0.23
GO:0010941~regulation of cell death	1.87	0.25
GO:0042981~regulation of apoptosis	1.85	0.39
GO:0042254~ribosome biogenesis	3.36	2.73
GO:0006917~induction of apoptosis	2.21	4.20
GO:0012502~induction of programmed cell death	2.21	4.32

*oxFO* oxidized fish oil, *FO* fish oil, *BP* biological processes, *FDR* false discovery rate

## Discussion

In the present study, the consumption of oxFO for 7 weeks did not alter the transcriptome profile in PBMCs in healthy subjects when compared to the intake of high-quality FO in a randomized controlled intervention study. Within-group analyses revealed a more prominent effect after intake of high-quality FO as more gene transcripts were changed from the baseline than within the oxFO group. The current study is to our knowledge the first to investigate effects at a molecular level after the intake of oxFO using high-throughput sensitive transcriptome analyses.

The intake of lipid oxidation products may disturb the normal redox state of the cell, alter normal cell function and signaling [7, 16], and ultimately lead to modulation of gene expression [39]. In a previous study with mice, the intake of moderately oxidized n-3 PUFA for 8 weeks induced oxidative stress in the intestine and enhanced plasma inflammatory markers and gene expression analyses showed an increase in the glucose-regulated protein 78 (GRP78) and glutathione peroxidase 2 (GPx2) mRNA [20]. We have previously not been able to detect changes in markers of oxidative stress such as urinary 8-iso-PGF2a, plasma a-tocopherol, plasma 4-hydroxy-2-hexenal and plasma 4-hydroxy-2-nonenal or glutathione, CAT activity, and GPx activity in erythrocytes or inflammatory markers in the same study after intake of oxFO compared to high-quality FO [27, 28]. Furthermore, the intake of oxFO, containing lipid oxidation products, did not alter biological processes or gene sets related to oxidative stress when compared to fish oil in the current study.

When the groups were analyzed separately, the mRNA level of BAK1 was significantly increased from baseline within the oxFO group. The BAK1 protein is localized to the mitochondria and functions to induce apoptosis. This protein also interacts with the tumor suppressor p53 after exposure to cellular stress [40], and the increased BAK1 mRNA level could be caused by the intake of lipid oxidation products in the oxFO group. BAK and BAX were recently shown to be involved in eicosanoid metabolism independent of apoptotic functions [41]. The increase in BAK1 mRNA may therefore be related to the eicosanoid precursor EPA and DHA, presented in equal amounts in the two fish oils. BAK1 mRNA was also increased from the baseline within the FO group, although not significantly (Table 4), and biological processes related to apoptosis were regulated within both fish oil groups.

Biological processes related to translation elongation, translation, and apoptosis were enriched within both groups, while biological processes related to cell cycle was only enriched within the oxFO group. However, we have previously shown that the intake of high-quality FO regulated gene transcripts related to apoptosis, cell cycle, and ER stress when compared to the intake of high-oleic sunflower oil [36]. Our data is in accordance with Bouwens et al.’s, who also demonstrated that the expression of genes involved in pathways related to processes such as transcription, translation, and cell cycle were regulated in PBMCs after the intake of FO (1.8 g/day EPA+DHA) for 26 weeks in elderly subjects [31]. Thus, processes related to the translation, apoptosis, and cell cycle may be modulated after the intake of fish oil independent of oxidative status. These processes and pathways are involved in normal cellular functions and may ultimately influence whole body health. We did not detect any regulated pathways or processes related to lipid metabolism, antioxidant defense, and inflammation as previously reported after n-3 supplementation [31, 42–44]. This could be explained by the inclusion of healthy subjects in the current study, and the results may be different in subjects with elevated levels of inflammation, oxidative stress, or serum lipids.

CD55 was downregulated from the baseline within both groups and independent of the oxidative status of the fish oils. CD55 is part of the complement system and has been suggested to play a role in the pathogenesis of atherosclerosis [45]. CD55 deficiency has also been shown to protect apoE^−/−^ micefrom atherosclerosis [46]. The downregulation of CD55 mRNA by fish oil may thus be one of the mechanisms by which fish oil can exert its beneficial effect.

The changes in the oxidative status of the oil as measured by increased PV and AV occur prior to a measurable reduction in the concentration of fatty acids. Thus, the oxidized and the non-oxidized FO contained an equal amount of fatty acids, including n-3 fatty acids (Table 1). The similar transcriptome profile may therefore be explained by the similar fatty acid profile of the FOs [28] as they originated from the same batch.

Conflicting results related to the prevention of cardiovascular diseases by marine LCPUFAs have recently been published [47]. It has been suggested that the oxidation of the marine oils may be responsible for the lack of effect [15]. A recent study investigating the effect of omega-3 supplements with different oxidation level on circulating lipid parameters showed a reduction in plasma triglycerides independent of the oxidative status of the fish oil. However, a beneficial effect on plasma total cholesterol was only evident in the group consuming less oxidized fish oil [48]. In the current study, a more prominent effect was evident after the intake of FO as 11 gene transcripts were significantly (FDR < 0.1) changed from baseline whereas 3 within the oxFO group (Table 4). Whether this is related to the oxidative status of the fish oils is currently not known and needs further investigations.

The present study was originally designed to investigate the effect of oxFO on the circulating markers of oxidative stress, inflammation, and plasma lipids compared to high-quality FO, and the power of the study was calculated based on the relative change in plasma n-3 compared to the high-oleic sunflower oil [28]. We cannot rule out the possibility that the current study lacks the power to detect differences between the two FO groups when it comes to the transcriptome profile. However, transcriptome analyses in PBMCs represent a sensitive high-throughput method to detect changes caused by diet [30]. To increase the power in the current study, we included analyses where genes were grouped based on involvement in the same biological process, pathway, or network.

The strengths of the present study are that the study design was performed as a blinded randomized controlled trial with high compliance, as described in more detail elsewhere [28]. The transcriptome analyses have been performed with an exploratory approach where the identification of regulated gene transcripts, biological processes, and networks was done with several data analysis strategies. The major limitation is the relatively low daily dose of oxidized lipids (PV/AV) administered and the relatively short intervention period. The study group included healthy subjects, and we cannot rule out that by using another study group more susceptible to the intake of oxFO, this would have led to different results. There is also a chance that other cellular compartments or tissues such as the intestine may have been affected by the intake of oxFO in the present study.

## Conclusion

By using a transcriptome approach, we aimed to investigate the effects at the molecular level prior to changes in circulating markers. The results demonstrate that the short-term consumption of oxidized fish oil in healthy subjects did not alter the transcriptome profile in PBMCs compared to the intake of high-quality FO. Whether these results are applicable for other oxidized marine oils, longer interventions, or valid in subjects with elevated levels of inflammation and oxidative stress needs to be addressed.

## Additional files

Additional file 1: Table S1. Significantly changed gene transcripts (*p* > 0.05) between the oxFO and FO groups determined by a moderated *t* test (Limma), by comparing the relative change from the baseline to 7 weeks of intervention. *oxFO* oxidized fish oil, *FO* fish oil, *FC* fold change from baseline, *FDR* false discovery rate. (XLSX 34 kb) Additional file 2: Table S2. GSEA analyses applied on the genes defined as expressed in PBMCs (21,236) for comparison of the transcriptome profile between the two groups oxFO vs FO. Gene sets in the C2 cp collection were used. *ES* enrichment score. *NES* normalized enrichment score. (XLSX 33 kb) Additional file 3: Table S3. GSEA analyses applied on the genes defined as expressed in PBMCs (21,236) for comparison of the transcriptome profile between the two groups oxFO vs FO. Gene sets in the C5 bp collection were used. *ES* enrichment score, *NES* normalized enrichment score. (XLSX 25 kb)

## Abbreviations

AV: anisidine value
CHD: coronary heart disease
FC: fold change
FDR: false discovery rate
FO: fish oil
LCPUFAs: long-chain polyunsaturated fatty acids
meq/kg: milliequivalents per kilogram
oxFO: oxidized fish oil
PBMCs: peripheral blood mononuclear cells
PV: peroxide value
